# Psychiatric care and education understood from a student perspective: Enhancing competences empowering personal and social recovery

**DOI:** 10.1111/scs.13097

**Published:** 2022-06-10

**Authors:** Janne Brammer Damsgaard, Camilla Margrethe Lyhne Overgaard, Dorte Wiwe Dürr, Anita Lunde, Peter Thybo, Regner Birkelund

**Affiliations:** ^1^ Department of Public Health, Nursing Aarhus University Aarhus Denmark; ^2^ Psychiatric Research Unit, Central Denmark Region Herning Denmark; ^3^ Department of Nursing Via University College Horsens Denmark; ^4^ University Hospital of Southern Denmark Vejle Denmark; ^5^ Department of Regional Health Research, University of Southern Denmark Odense Denmark

**Keywords:** biomedical approach, competence, education, presence, psychiatric care, role model

## Abstract

**Background:**

During the last decades, a recovery‐based approach has called for a change in mental health care services. Several programmes have been presented, and the need to develop student and professional competences in education and clinical practice has been documented.

**Aim:**

The aim of this study was to explore how psychiatric care is understood seen from a student perspective (nursing students, masters nurses and a master in applied philosophy) with focus on their personal competences and the educational interventions empowering processes for users' personal and social recovery.

**Method:**

A qualitative design with a phenomenological–hermeneutic approach based on the French philosopher Paul Ricoeur's theory of interpretation. Data were collected through semi‐structured interviews.

**Findings:**

All interviewees expressed that both theoretically and clinically students did not experience a recovery‐oriented approach empowering users' personal and social recovery process. On the contrary, they experienced that both education and practice were dominated by a biomedical approach providing clinical recovery. However, several students were aware of their need of developing personal and relational competences to be able to support the users' personal and social recovery journey. The students expressed that there is a need for educational processes targeting personal competences in ‘becoming a professional’ supporting ‘presentness and awareness’ and thereby the development of relational abilities and the courage to engage.

The results relate to two nursing schools and two universities.

**Conclusion:**

A biomedical approach dominates and makes it difficult to develop students' personal competences during education in practice and theory vital to the development of personal and social recovery‐oriented practices. It is recommended that educators—in practice and in school—accentuate presentness, awareness and creativity as crucial relational capabilities and incorporate this in their teaching and supervision method, supporting the education and formation of the students' (and teachers' and supervisors') personal development processes.

## BACKGROUND

During the last decades, a recovery‐based approach has called for a change in health care. Several programmes have been developed in order to implement a recovery‐based approach, and challenges to develop student and professional skills and competences in education and clinical practice have been documented (1, 2, 3, 4, 5, 6, 7). This article contributes with knowledge to the psychiatric practice and educational curricula exploring how psychiatric care is understood seen from a student perspective. There will be focus on personal competences and educational interventions.

The recovery‐based approach is illuminating more focus on users' autonomy and personal values. The relation between user and professional is moving away from understanding the professional as an expert to the professional being *available* supporting the person on his or her recovery journey (8, 9). A systematic review and meta‐analysis of recovery educational interventions for mental healthcare professionals by Eiroa‐Orosa and colleagues (10) shows that when aiming at recovery there is not ‘one unique truth’ to be predicted by professionals. This understanding is creating a need for new competences where reflection on practice is focal and different educational strategies seek to facilitate empowerment to achieve full citizenship of mental health users by involving users and private as well as social contexts such as family, friends and leisure activities (10). However, according to Eiroa‐Orosa, recovery and empowerment training activities seem to have a clear but moderate impact on the beliefs and attitudes of mental healthcare professionals. Qualitative studies point in the direction of organisational and educational obstacles (11, 12, 13, 14, 15). It appears that the healthcare professionals at psychiatric wards do have *knowledge* about ‘recovery’ and a ‘recovery‐oriented approach’, and they do have *intentions* about integrating this in their daily work. Even so, the healthcare professionals have difficulties with establishing *consensus* about what to understand by recovery and to put their *knowledge to work in practice*. Similarly, Shepherd and Slade (9) conclude, that ‘We still believe that the greatest challenge for recovery ideas is translating fine rhetoric into tangible changes “on the ground”’ (16 p. 4).

The knowledge of nursing students' recovery attitudes and education in psychiatry are sparse (17). However, in Australia, challenges with implementing personal and social recovery in recovery‐focused services have been ascertained (18). Therefore, the education of health professionals has been identified as a major strategy. A qualitative study aimed to evaluate Australian students' views and opinions on having been taught ‘recovery in mental health nursing’ by a person with a lived experience of significant mental health challenges. Two main themes were identified: ‘looking through fresh eyes’—what it means to have a mental illness; and ‘it's all about the teaching’ (18). Being taught by a person with a lived experience was considered integral to the learning process and the students found that this innovative teaching approach could enhance consumer participation and recovery‐focused care (18).

Another qualitative study by Demir et al. (19) explored student nurses' clinical experiences during their first clinical contacts with psychiatric patients. It was found that ‘empathy’ and the ‘development of relationships’ reduced stigma towards the end of their clinical practice. The students stated that communication and psychiatric clinical practice helped them develop interpersonal competences. The possibility of promoting empathy to nursing students in a psychiatric setting through creative reflective teaching strategy was also the focus in a mixed‐method study by Webster (20).

Creativity and the learning of communication is also mentioned in a qualitative study by Azevedo et al. (21). The objective was to analyse ‘the sensed perception’ of nursing students in the learning of communication in a psychiatric hospital, recording remarkable experiences at the end of a practical activity (21). It was concluded that the learning of communication was significant, making ‘the psychiatric hospital a space to listen to what is inside’ (21).

In this educative perspective, a Swedish paper argues (22) that there is potential in psychiatric and mental health nursing as a caring, reflective and therapeutic practice promoting recovery. Psychiatric and mental health nursing provides *a transformative force* contributing to incorporating person‐centred values and practices in health care in general (22).

The field of psychiatric care and education is complex, hence to Borg and Karlsson, understanding and implementing a recovery‐orientated culture within psychiatric healthcare, it is not ‘enough’ to focus on *communicating theory and practice* (23) but moreover to sense, reflect on and develop students' and professionals' personal skills and competences understanding themselves and the culture which they are a part of. These perspectives are also highlighted in the study by Damsgaard and Phoenix (24). In concordance with this, a literature review and documentary analysis on recovery training in mental health practice by Campbell and Gallagher (25) shows that for this to be developed, relational aspects will need to be considered both within education and implementation to clinical practice. For example, Campbell and Gallagher find that by relating and listening to users' stories of lived experience, nurses (and students) can adjust the focus of their work, learning from users, relating to them, meeting their needs, hopes and dreams.

This is also a focal point of attention in the recommendations of The Danish Society of Psychosocial Rehabilitation (DSPR) which point out that the curriculum at the Danish educational institutions is predominantly based on biomedical knowledge (26). DSPR advocate for an introduction of pluralism in the curriculum for psychiatric education. To DSPR, it is a question about encouraging and developing the ability of the employees and students to hope, be creative, caring for and showing compassion, be realistic and to develop resilience based on explicitations of life stories and thereby individual reasons for psychological challenges. This focus is important because it is the basis for all further efforts (26).

Within this perspective, allowance must be made for empowering the healthcare professionals and students in their personal and educational formation, developing their professional focus and involvement. As documented above, this educational focus and task is still mostly described seen from view of other parties than the students themselves. There is a need for a comprehensive understanding of what *students* experience as challenges and what they point out as worthy of attention.

## AIM

The aim of this study was to explore how psychiatric care is understood seen from a student perspective (bachelor nurse students, masters nurses and a master in applied philosophy) with focus on their personal competences and the educational interventions empowering processes for users' personal and social recovery.

## METHODS

### Design

Within a phenomenological–hermeneutic approach, a qualitative design was chosen. The study involved semi‐structured interviews (27) and applied Paul Ricoeur's phenomenological–hermeneutic theory of interpretation in processing the collected data (28, 29) consisting of a naïve reading of data, a structural analysis and a comprehensive understanding.

### Preunderstanding

In the present study, preunderstandings were appraised through self‐reflection, self‐awareness and by being observant, attentive and sensitive to the world of experience. Being aware of power relations, positions and opinions working as professionals connected with psychiatric care, the aim was that the students' lifeworld should present itself in its complexity (through interviews and interpretation), seeing what was well known in a new light. This attitude was also adopted in the analysis process. However, it is probably not possible to be fully aware of our preunderstandings—‘preunderstandings lie deep’ (30, p. 136), but confronted by what was perceived as new and strange, the researcher's own preunderstandings were recurrently illuminated.

### Participants and data collection

Given the purposive strategy of simultaneously recruitning participants with experience from both theoretical and clinical teaching in psychiatric care and representing three different settings (two universities, a nursing school and a clinical practice), it was possible to include seven students (four bachelor nurse students, two masters nurses and a master in applied philosophy) between the ages of 23 to 49—see Table 1.

**TABLE 1:** Study participants

Participant	Gender	Educational level
A	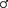	Masters student Registered Physiotherapist (RP)
B	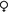	Masters student Registered Nurse (RN)
C	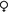	Masters student (RN)
D	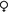	Nursing student 6th sem.
E	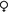	Nursing student 6th sem.
F	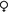	Nursing Student 6th sem.
G	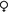	Nursing student 7th sem.

The interviews and data analysis were performed by the five authors from June to October 2021. In total, three interviews were performed as face to face interviews, while four interviews took place online. A semi‐structured interview guide with open questions was used (27)—see Table 2. To achieve openness in the interviews, the participants were asked broad and open questions through which they were asked to describe and reflect on their immediate thoughts about the received and experienced education in psychiatric care describing which competences the informants found important in relation to psychiatric care. During the interviews, the participants' reflections directed the interviews, but we specifically strived to focus on experiences related to theoretical and clinical learning situations, exploring how psychiatric care is understood and focusing on personal competences and educational interventions. The interviews lasted between 45 and 60 min and were subsequently transcribed verbatim.

**TABLE 2:** Semi‐structured interview guide

Question 1: Tell me your immediate thoughts about your received education in psychiatric care.
Question 2: Which competences are important in psychiatric care in relation to empower processes towards a meaningful life for the patient?
Question 3: How do these competences manifest themselves in practice?
Question 4: How do we develop these competences?

### Ethical considerations

All participants were informed both verbally and in writing about the purpose of the project. They were assured that participation was voluntary, that they would be able to withdraw from the project at any time, and that all data would be made anonymous (Declaration of Helsinki, 1964). According to Danish law, approval from the Regional Committee for Medical Research was not required because of the non‐biomedical character of the study. The project was approved by and is registered at VIA University College and Central Denmark Region in accordance with the provision of General Data Protection Regulation (GDPR). Data were processed and stored according to the recommendations of VIA University College and Central Denmark Region, and a shared collaboration agreement in relation to shared data responsibility was formulated.

### Data analysis and interpretation

Inspired by Ricoeur's theory of interpretation, we conducted a three‐level interpretation process to reveal the meaning of the participants' experiences (31). The process included the phases—naïve reading, structural analysis and comprehensive understanding. According to Ricoeur, this method benefits from the dialectical movement between explanation and understanding and provides an understanding of what the text as whole addresses (32). In the naïve reading, the text was read several times and with as open a mind as possible to achieve an initial understanding of what it was all about. Ricoeur emphasises that this phase is important but must be validated by subsequent structural analysis. In the structural analysis, we structured and explained the text by units of meaning (what is said) and units of significance (what the text speaks about). This allowed us to achieve a deeper understanding of the text, creating themes and subthemes—see Table 3. The last level of interpretation was conducted as a comprehensive understanding that entailed revising, broadening and deepening the awareness through critical reflection (31). The themes derived from the texts in the structural analysis became the basis of the comprehensive understanding (31). Based on the findings relevant theoretical perspectives focusing on formation, sensitivity, presence, role models and existing research were included to achieve new insight, thus creating new knowledge about the students' experiences.

**TABLE 3:** Example of the analysis process—From quote to theme

Meaning units ‘What is said’	Units of significance ‘What the text speaks about’	Subthemes	Themes
*‘There was a lot of teaching about the different diseases and what the different symptoms were and what you could be aware of if you had a patient with for example schizophrenia’*	The structures in the healthcare system are supporting the biomedical paradigm. The professional language retains the patients in their disease and creates a gloomy and pessimistic approach	Biomedical approach	Experiences meeting practice—uncertainty of psychiatric care
*‘If I am in a medical unit*, *well then I know the things I have to do. I did not know what to do (at the psychiatric unit)’*	Psychiatric care is experienced to be very different from somatic care; the somatic care is associated with being busy and handling instrumental tasks at a certain pace. It is experienced that psychiatric care is indefinable, and it is difficult navigating in what psychiatric care is all about and how it can be learned	Psychiatric care	
*‘Of course*, *I am (training to be) a nurse*, *but I do not hang my personality in the changing room at work. I use it*, *and that's okay’*	The students feel the relation between them and ‘the other’ is depending on *being present* with the patient. Being present is recognisable and sensed, when the students experience these qualities following an authentic and experienced supervisor—a role model. The students are reminded of such sensations when their human qualities and values are put at stake in the psychiatric care	Personal competences Relational care	Experiences engaging in practice—daring to use yourself

## FINDINGS

### Naïve reading

The naïve reading of the interviews showed that the informants experienced that both the theoretical and clinical teaching in psychiatric care had a biomedical approach. They had difficulty defining what psychiatric care was about, and they experienced challenges and dilemmas of using themselves when caring for people with mental illness.

### Structural analysis

#### Experiences meeting practice—uncertainty of psychiatric care

Across the interviews, it is obvious that the students in both theory and practice experience that teaching and learning was guided by a biomedical approach, as one of the student stated:

*‘There was a lot of teaching about the different diseases and what the different symptoms were and what you should be aware of if you had a patient with for example schizophrenia’* (B)

The students experience how structures in the healthcare system are supporting the biomedical paradigm. The professional language affects the patients.

*‘You were the expert*, *telling the patients that*, *this symptom*, *is because you are sick in your brain’* (G)

The students reflect on such experiences in the interviews and argue that the biomedical approach frames the approach to the patients:

*‘I feel that we create a ‘disease‐identity’ and retain people in it’* (C)

This statement illustrates how the students experience that patients are identified with their diagnosis. The students learn how to observe, treat and care for the patients based on this logic. For the masters students, this understanding becomes even clearer and states how the person and the person's life, before and next to the disease, are overlooked.

In the interviews, the students express that psychiatric care is experienced as very different from care in a somatic field, in the sense that somatic care is associated with being busy handling instrumental tasks at a certain pace. Psychiatric care is experienced indefinable and the students have difficulty understanding what psychiatric care is all about and how it can be learned.

*‘If I am in a medical unit*, *well then I know the things I have to do. I didn't know what to do when I came to the psychiatric unit’* (E)

In addition to the fact that the students experience psychiatric care difficult to define, they also notice a shift or change in care perspectives as the job was to be with patients without having instrumental tasks.

#### Experiences engaging in practice—daring to use yourself

Listening to the interviewees, it appears that students achieve important learning about psychiatric care when putting their own personal competences at risk. This insight transcends theoretical skills and is described as a silent, intuitive and existential capability. One student explained it like this:

*‘… when you meet another person*, *there is just something beyond it. Something beyond what you can describe or express with words…’* (C)

In different ways, the students express how using themselves in a caregiving situation, takes courage. A student stated it this way:

*‘I think it is important to support and acknowledge. Acknowledge the students when they put their own selves at risk’* (A)

From several angles, the students described their thoughts about using themselves when caring for people with mental health challenges. Reflecting and being aware of their own identity, they highlighted the importance of having role models to look up to. A student described it in the following way:

*‘I think you have to experience it*, *and be a part of it. It is a bit like apprenticeship*, *because you have to see how other professionals act in a situation*, *and how it works. If I try the same*, *does it work for me too or do I have to adjust a bit?’* (G)

Such experiences indicate that human intimacy is difficult to learn from the distance of a book chapter. It must be felt (experienced) in the relation. One student points this out:

*‘… it is not just about talking for an hour. It is all about your way*, *your approach to meeting this person that makes a difference’* (C)

## COMPREHENSIVE UNDERSTANDING

### Uncertainty of psychiatric care

Throughout the interviews the students experience that psychiatric care is dominated by a biomedical approach, which they find represented in both theoretical and clinical teaching. Based on the theoretical teaching, they have a hard time imagining what a psychiatric practice includes and this question remains in the clinic. Within practice, it becomes clear that the students are unsure of what psychiatric nursing really is. The more unknown relational and expressive tasks make them insecure and the students express difficulty defining what psychiatric care is. They clearly feel how they must use themselves in new ways, learning how to interact when confronted with unknown situations and insecurity.

Researcher in education Lars Geer Hammershøj explains how formation (‘bildung’) of character is an important element in education. Formation, ‘bildung’, implies development of a personal unique human capacity nessesary for the profession. To Hammershøj *education* is the acquisition of knowledge, skills and competences which constitutes an important foundation. *Formation* of character is the way you relate to yourself, to others, to the world and to the tasks you have to solve (Hammershøj, 2017). Following Hammershøj, the purpose of education is to develop ways of behaving and to develop a change in one's attitude, gaining new values. Moreover, it is about strengthening one's judgement, to be creative and open to new ways of understanding. Formation—the ‘bildung’—of students' character goes along with education and is motivated by a willingness to make a difference for the profession. To Hammershøj, the teacher plays an important role in the students' personal development process. In addition to imparting in theoretical education, the teacher must open new worlds to the student in a human and thereby professional direction.

Based upon this understanding of education, nursing schools should include personal and experience‐based activities in regard to the students' professional development. The students' education is dominated by ‘knowledge about’, for example psychiatric diseases where the biomedical paradigm dominates. However, in the psychiatric encounter the students really experience how educational elements such as knowledge and skills are not sufficient. Meeting psychiatric patients, values, ethics and attitudes become crucial, but the education has not prepared the students for this meeting.

The interviews show how the students have great willingness to learn ‘how to be’ a professional. With Hammershøj's understanding of higher education, both theoretically and clinically teaching needs to include the educational and formative purpose of psychiatric care. This means that the profession needs to, explicitly, state this in teaching as well as in various educational documents. Within this understanding the teacher has an important role in the students' development process towards becoming a professional. The students need to be trained, not only with knowledge and skills, but also with personal competences to act in new and unknown situations, empowering a basis for promoting personal and social recovery to the users. The interviews indicate that the students experience that they, more or less, are left alone with this task.

### Daring to use yourself

Throughout the interviews a focus on the importance of daring and acting on personal and relational momentary senses and events, emerges. Such aspects are described by the students as key moments of change and transcend theoretical skills. The awareness and personal competences are described as intuitive capabilities which originate in existential experiences and are expressed as ‘relational moves’ and joint subtle experiences such as a spoken phrase, a silence, a gesture, a shift in posture or a facial expression. Such events are, however, described by the students as ‘silent’ (tacit knowledge) and therefore particularly important for the supervisors and the health care professionals to articulate, mirror in their practice, and acknowledge as a vital and appreciated competence.

Within this context the students' relational experiences of ‘*something beyond what you can describe or express with words*’ are vital and are pointing to important aspects when interacting with the other person (the user). Moreover, it is understandable that students can feel at risk of being personally exposed when moving into this intersubjective moment of meeting. They are in need of acknowledgement and guidance regarding the potential of the present moment as an important gateway to creating a relationship and shared mental experiences with possible change. Within this process it is vital that schools and practice embrace and grapple with presence and awareness of our lived experiences and how to include and explore these same lived experiences (and intuition), that is including them, interacting in a relation empowering users' personal and social recovery.

In his research, psychologist Daniel Stern pinpoints small momentary events as events that ‘make up our worlds of experience’ (33, p. xi). He is interested in such moments as they are shared between two people. To Stern such lived experiences make up key moments of change and are the nodal points in our everyday intimate relationships. His focus is not about ‘meaning’ in the usual clinical sense, but about explaining the present (in terms of the past), establishing associative linkages that are interpretable. It is about experience as it is lived. Through his studies, based upon video sequences of mother‐infant interaction, he has shown how much occurs in a moment that lasts only seconds (34). To Stern, these moments are *basic building blocks of experience*. It is about ‘the subjective experience’ and especially what leads to change (33 p. xiii). In and of itself, verbally understanding, explaining or narrating something is not sufficient to bring change. To Stern there must also be an actual experience, ‘a subjectively lived happening’. An event must be *lived*, with feelings and actions taking place in real time, in the real world, with real people, in a moment of *presentness*. The present moment is the moment of subjective experience as it is occurring, and the first step towards understanding experience is therefore to explore and understand the present moment.

Although arguing to work personal and social recovery‐oriented, this study shows that the students experience psychiatric health care and education as dominated by a biomedical approach promoting clinical recovery. They struggle with understanding what psychiatric care is all about and are insecure when they have to engage in existential and relational interactions with the patients. The instrumental tasks are described as easier to turn to. However, several of the students are aware that they are in need of role models and more knowledge in regard to developing their personal and relational competences, involving both educational and clinical practice. This aligns with what Etienne Wenger describes as ‘access to experience in order to feel connected to a subject of matter’ (35, 36). Being an active practitioner with an authentic form of participation might be one of the most deeply essential requirements for teaching.

But the discrepancy between what the professionals and students know and what they practice represents ‘a challenge related to knowledge’ (35). To Wenger ‘knowledge can be more or less explicit; learning can be more or less formal; an impression can be more or less conscious; a meaning can be more or less individual’ (35 p. 66). However, the dimensions interact; they do not define a spectrum. According to Wenger there can be both ‘intense participation and intense reification—an excessive emphasis on formalism without corresponding levels of participation, or conversely a neglect of explanations and formal structure, can easily result in an experience of meaningslessness’ (35 p. 67). This complexity is worth including in regard to understanding and creating knowledge that resonates with the students, the professionals and the users.

## DISCUSSION

Our study found that there is a need for educational processes (in both practice and school) targeting personal competences in ‘becoming a professional’ supporting ‘presentness and awareness’ and thereby the development of relational abilities and the courage to engage in them. This is similar to Moustakas' findings focusing on the challenges of facilitating growth of individuality and relationships (37). He find that there is ‘meaning present in creating new ways of being and relating’ (37) and he is targeting three perspectives: ‘*Being In’;* focusing on being aware and understanding from the vantage point of the other; ‘*Being For’;* taking a stand, being in collusion, listening; and ‘*Being With’;* being present as an individual self with knowledge and experience, indwelling, keeping alive own imagination, immersing oneself into the other's account of an experience.

Our study shows that understanding psychiatric healthcare within this personal and relational perspective, practice and education needs to facilitate more than theoretical knowledge and skills. This aligns with the study by Byrne et al. ‘Things you can't learn from books': Teaching recovery from a lived experience perspective’ (18). The importance of drawing on more than theory, is also found in the study by Dickens et al. (38) showing the relevance of an authentic voice of expertise‐by‐experience incorporated into psychiatric education about individuals diagnosed with borderline personality disorder.

To sum up, aspects like values, ethics and attitudes become important and need to be challenged and discussed, enhacing competences such as awareness, intuition, imagination, creativity, good judgement and courage, and this also involves the teachers´ competences and their curricula. This is in line with sociologist Hartmut Rosa's research arguing that ‘school not only opens or closes individual axes of resonance, but forms the quality of our relationship to the world as a whole´ (39). As an example of resonance pedagogy he refers to the movie ‘Dead Poets Society’. Here, the teacher–student relation is depicted, illustrating how, for example, lyrics begin to ‘speak’ to the students, creating personal and social changes (40). As a teacher and a healthcare professional, awareness of, and inclusion of such aspects, are crucial. Based on this understanding, the teacher and the supervisor play an important role in the education and formation process of the student. In addition to imparting knowledge the teacher and supervisor must open ‘new worlds' to the student in both a personal and professional direction.

### Methodological considerations and study limitations

This study was based on interviews with seven interviewees (students) with a mixed background (bachelor nurse students, masters nurses and a master in applied philosophy) and all related to psychiatric health care. Seven participants are a small number but since the aim of qualitative studies is to gain a deeper insight into the students´ personal experiences, it is irrelevant to discuss the number of participants, hence more participants could have added data to this study. It is, however, relevant whether the selected participants can redeem the problem under study (41, 42). It was assessed within the research group that the participants were all suited to enter the study because of their interest and reflections on the field. As most of the participants were recruited by their teachers, we were especially attentive to the power relations during the interviews, trying to be aware of, for example tone and body language. It was not our intention to generate generalisable knowledge using the phenomenological–hermeneutic method to discover what psychiatric care and education *is* like, only what this *may* be like. Neither was it our intention to test the evidence of applied theories, but to discuss the themes that arose from the participants' stories, being aware that the course of each student will always be unique.

## CONCLUSION AND IMPLICATION FOR PRACTICE

This qualitative study shows how students experience psychiatric healthcare education in theory as well as in practice. The students find that a biomedical approach with a focus on pathology and clinical recovery dominates their education. This causes uncertainty in clinical practice. The students are insecure in regard to understaning what psychiatric nursing is all about. To be able to meet another person with a unique life story that precedes the mental illness and disorder requires the ability to build a good relationship. It is a relationship that is primarily based on the professional's personal competences and human knowledge such as the ability to meet the other’ in the present moment, empathy and deep understanding of the other's life situation. It takes good judgement, intuition, creativity and the courage to go new ways. The students find that formation of character as a part of their education is overlooked in both theory and practice. However, they find great inspiration and support when they experience professional role models unfolding their human and tacit knowledge in practice and in theory.

Following the students' perspectives there are several ways to enhance personal competences, for example joining supervision, incorporating patient stories within practice, including creative and imaginative educational elements—for example reflecting on paintings, photographs or literature. They suggest that this can promote the ability and courage to seize the present moment, getting conscious of the mind of our self and the other.

### AUTHOR CONTRIBUTION

Each author has made substantial contributions to the conception or design of the work and has approved the submitted version and agrees to be personally accountable for the author's own contributions and for ensuring that questions related to the accuracy or integrity of any part of the work, even ones in which the author was not personally involved, are appropriately investigated, resolved and documented in the literature. All authors have read and agreed to the published version of the manuscript.

### CONFLICT OF INTEREST

The authors declared no potential conflicts of interest with respect to the research, authorship and/or publication of this article.
